# Monitoring of age- and gender-related alterations of endocannabinoid levels in selected brain regions with the use of SPME probes

**DOI:** 10.1007/s11306-023-02007-9

**Published:** 2023-04-12

**Authors:** Anna Roszkowska, Ilona Klejbor, Joanna Bogusiewicz, Alina Plenis, Barbara Bojko, Katarzyna Kowalik, Janusz Moryś, Tomasz Bączek

**Affiliations:** 1 Department of Pharmaceutical Chemistry, Medical University of Gdańsk, Gdańsk, Poland; 2 Department of Anatomy, Institute of Medical Sciences, Jan Kochanowski University, Kielce, Poland; 3 Department of Pharmacodynamics and Molecular Pharmacology, Collegium Medicum in Bydgoszcz, Nicolaus Copernicus University in Toruń, Bydgoszcz, Poland; 4 Department of Analytical Chemistry, Medical University of Gdańsk, Gdańsk, Poland; 5 Department of Normal Anatomy, Pomeranian Medical University, Szczecin, Poland

**Keywords:** Endocannabinoids, Brain sampling, Solid-phase microextraction, LC–MS, MS

## Abstract

**Introduction:**

The endocannabinoid system consists of different types of receptors, enzymes and endocannabinoids (ECs), which are involved in several physiological processes, but also play important role in the development and progression of central nervous system disorders.

**Objectives:**

The purpose of this study was to apply precise and sensitive methodology for monitoring of four ECs, namely anandamide (AEA), 2-arachidonoyl glycerol (2-AG), N-arachidonoyl dopamine (NADA), 2-arachidonyl glyceryl ether (2-AGe) in selected brain regions of female and male rats at different stages of development (young, adult and old).

**Methods:**

Biocompatible solid-phase microextraction (SPME) probes were introduced into the intact (non-homogenized) brain structures for isolation of four ECs, and the extracts were subjected to LC–MS/MS analysis. Two chemometric approaches, namely hierarchical cluster analysis (HCA) and Principal Component Analysis (PCA) were applied to provide more information about the levels of 2-AG and AEA in different brain structures.

**Results:**

2-AG and AEA were extracted and could be quantified in each brain region; the level of 2-AG was significantly higher in comparison to the level of AEA. Two highly unstable ECs, NADA and 2-AGe, were captured by SPME probes from intact brain samples for the first time.

**Conclusion:**

SPME probes were able to isolate highly unstable endogenous compounds from intact tissue, and provided new tools for precise analysis of the level and distribution of ECs in different brain regions. Monitoring of ECs in brain samples is important not only in physiological conditions, but also may contribute to better understanding of the functioning of the endocannabinoid system in various disorders.

**Supplementary Information:**

The online version contains supplementary material available at 10.1007/s11306-023-02007-9.

## Introduction

The endocannabinoid system (ECS) is an integral component of body homeostasis responsible for the regulation of numerous physiological processes, including energy metabolism, immune response, motor activity and pain perception (Silvestri & Di Marzo, 2013). The first studies on this system were reported in 1992 with the discovery of endogenous molecule—N-arachidonoylethanolamide (anadamide, AEA) in pork brain samples (Devane et al., 1992). The ECS is composed of different types of receptors, including cannabinoid receptors, i.e. CB1 and CB2 and transient receptor potential cation channel subfamily V member 1 (TPRV1), and also endogenous ligands (i.e., endocannabinoids, ECs) and different enzymes (Maccarrone, 2020). In the central nervous systems (CNS), the ECS affects thermoregulation, appetite, memory processes, mood and psychoactive activities. Dysregulation of this system is observed in CNS disorders, such as Parkinson’s disease, epilepsy, depression and schizophrenia (Dickens et al., 2020; Marchioni et al., 2018; Smaga et al., 2017). It was also observed that in the brain, the ECS may be expressed in age-, gender-, and structure-dependent manner (Almeida et al., 2022; Gao et al., 2020; Pati et al., 2018; Piyanova et al., 2015).

The ECs are endogenous molecules derived from omega-6 polyunsaturated fatty acids, and, in contrast to other types of neurotransmitters, are mainly synthesized in the postsynaptic neurons. Once released into the synaptic space, they bind to CB1 receptors in the presynaptic membrane and inhibit the release of other neurotransmitters, such as noradrenaline and serotonin. Another feature that distinguishes ECs from other neurotransmitters is that those molecules are not stored in the CNS, but are synthesized “on-demand” and then immediately metabolized to different compounds, including arachidonic acid (Lu & MacKie, 2016). The ECs differ in structure, potency and affinity for cannabinoid receptors. The best known and well-studied ECs are AEA and 2-arachidonylglycerol (2-AG), and in the brain of rodents and humans the level of 2-AG is hundred to thousand times higher than the level of AEA (Almeida et al., 2022; Buczynski & Parsons, 2010; Zou & Kumar, 2018). Within CNS, 2-AG is primary endogenous ligand of CB1, while AEA also activates other receptors, such as TRPV1 and negatively regulates 2-AG synthesis in the striatum (Maccarrone et al., 2008). Over the last years, the growing interest in the functioning of ECs led to the discovery of multiple other compounds currently comprising families of N-acylethanolamides (e.g., AEA), N-acyldopamines (e.g., N-arachidonoyl dopamine, NADA) and arachidonic acid derivatives (e.g., 2-AG and 2-arachidonoyl glycerol ether, 2-AGe). However, little is known about the level and distribution of ECs-like compounds in biological samples and their role within CNS, which is mainly due to low stability of these molecules in biological matrices (Bobrich et al., 2020; Daniel Kratz et al., 2021). Therefore, determination of such unstable metabolites requires the use of very efficient sample preparation and highly sensitive analytical instruments (Gong et al., 2015; Marchioni et al., 2018).

Up to date, the level of ECs have been determined in various biological samples, however, blood and its fractions remain the most commonly chosen matrix for monitoring of the level of particular ECs (Acquaro Junior et al., 2019; Chu et al., 2020; Gachet et al., 2015; Gong et al., 2015; Gurke et al., 2019; Kirkwood et al., 2016; Luque-Córdoba et al., 2018; Marchioni et al., 2020; Ney et al., 2020), also referred as ‘circulating ECs ‘ (Coccaro et al., 2018; deRoon-Cassini et al., 2022; Dickens et al., 2020). In addition, other matrices, such as cerebrospinal fluid (Kantae et al., 2017), brain homogenates (Bobrich et al., 2020; Gong et al., 2015; Qi et al., 2015; Smaga et al., 2017) and intact brain (Aslam et al., 2019), hair (Behnke et al., 2021; Chu et al., 2020; Gao et al., 2020; Ney et al., 2020; Voegel et al., 2021), liver (Pati et al., 2018), and saliva (Ney et al., 2020) were also employed for trace analysis of selected ECs. In most of those studies, AEA and 2-AG were analyzed, however, the protocols for simultaneous analysis of different ECs were also reported.

Analysis of ECs and related compounds still constitutes a challenge owing to the diversity of biofluids and tissues, and also to the wide range of concentrations at which they can be found in biological matrices. Therefore, various analytical methods have been employed for the extraction and determination of ECs, and currently most methods are based on highly sensitive, efficient and selective liquid chromatography tandem mass spectrometry (LC–MS/MS) approaches (Marchioni et al., 2018). For the isolation of ECs from biological samples, liquid–liquid extraction (LLE) (Bobrich et al., 2020; Chu et al., 2020; Gong et al., 2015; Gurke et al., 2019; Kirkwood et al., 2016; Daniel Kratz et al., 2021; Smaga et al., 2017) and solid phase extraction (SPE) (Behnke et al., 2021; Gao et al., 2020; Luque-Córdoba et al., 2018) have been the most frequently used so far, however, simple protein precipitation (PPt) (Coccaro et al., 2018; Qi et al., 2015) or combination of few traditional techniques (Dickens et al., 2020; Gachet et al., 2015; D. Kratz et al., 2022; Ney et al., 2020) have been also reported. For many years, those traditional approaches of sample preparation have been applied for isolation of ECs from biological matrices, however, there is a considerable difficulty to monitor the level of ECs in brain tissue with the use of those techniques as prolonged extraction procedure, enzymatic activity and sample storage significantly affect the concentrations of those endogenous molecules (Buczynski & Parsons, 2010; Gurke et al., 2019; Daniel Kratz et al., 2021). Recently, modern microextraction technology, namely solid-phase microextraction (SPME) has been proposed for the isolation of AEA and 2-AG from biological samples (Acquaro Junior et al., 2019; Aslam et al., 2019; Oliveira et al., 2021; Souza et al., 2019). Indeed, the introduction of this microextraction technology to the bioanalysis of ECs has opened new possibilities in analytical protocols via shortening the analysis time and eliminating the errors related to sample handling, thus providing new tools for isolation of unstable compounds present at trace levels in the sample (Roszkowska, Yu, et al. 2018; Yu et al., 2020). SPME has shown great potential in the metabolomics studies as it facilitates the isolation of broad range of hydrophilic and hydrophobic low molecular weight molecules (100—1200 Da) from biological fluids as well as from intact (non-homogenized) semi-solid and solid tissues (Bojko et al., 2014; Reyes-Garcés et al., 2018). In addition, SPME sampling do not disturb the biochemical equilibrium in the system under study, which have been proven in various metabolomics and drug monitoring studies performed directly in the living system (in vivo SPME) (Bojko et al., 2021; Yu et al., 2022).

The purpose of this study was to apply modern SPME-based methodology coupled to LC–MS/MS for monitoring four ECs (AEA, 2-AG, NADA and 2-AGe) three intact brain structures. After optimization of microextraction conditions, 4 mm length biocompatible SPME probes were introduced into cerebellum, cortex and striatum for the sampling of ECs from young, adult and old female and male rats. Next, the autoscaled data sets describing the levels of 2-AG and AEA in three structures of rat brain were evaluated by the hierarchical cluster analysis (HCA) and Principal Component Analysis (PCA) for better understanding of the functioning of these structures in respect to the levels of ECs. PCA and HCA are based on different mathematical algorithms, but allow to illustrate in a graphic form the relationships between the variables and/or objects without losing any significant information (Granato et al. 2018). The output of HCA is a dendrogram, which shows the hierarchical relationship between the clusters. In the PCA, that belongs to a group of factor analysis, the number of variables in a data set is reduced by finding linear combination of those variables, which explain most of the data variability (Jolliffe 2002). Thus, PCA transforms original measured data into new uncorrelated (independent) variables called Principal Components (PCs), which are a linear combination of the original variables, and can be used for analysis of the relationships between the objects and the variables by founding trends, groupings or outliers on loadings and score plots. Overall, several factors were taken into account to enhance the stability of analytes and method performance so as to facilitate in the future in vivo quantification of trace and unstable ECs in the CNS in order to understand their role in the functioning of particular brain structures.

## Materials and methods

### Chemicals

Standards of AEA, 2-AG, NADA and 2-AGe were obtained from Merck (Darmstadt, Germany). Deuterated isotopologue of AEA—AEA-d_11_ (internal standard, IS) from Cayman Chemical (USA). LC–MS-grade methanol (MetOH), acetonitrile (ACN), isopropanol (IPA), formic acid (FA), acetic acid and ammonium acetate were supplied by Merck (Darmstadt, Germany). Water used in the experiments was purified with a milli-Q system (Molsheim, France). Standard stock solutions were prepared in MetOH. Biocompatible SPME probes with C18 coating were supplied by Merck (Darmstadt, Germany). A stock solution of ECs and IS were prepared in ethanol (EtOH) (Chempur, Poland) at a concentration of 1 mg/mL and 0.5 mg/mL, respectively. Working standard solutions of ECs were prepared by dissolving an appropriate volume of stock solution in the working solutions. A working standard solution of IS at a concentration of 100 µg/mL was prepared by dissolving an appropriate volume of IS stock solution in ACN/water/FA (80:20:0.1, *v/v/v*). The stock and working solutions of ECs and IS were stored at – 80 °C until use.

### Animals and brain tissue preparation

The experiments were performed on Wistar Han rats provided by Tri-City University Animal House—Research Service Centre. The animals were kept on a normal day-night cycle at 22 ± 2 °C with access to food and water ad libitum. For each analyzed group of rats (divided based on age and gender) 3 animals were used. Rats were sacrificed through decapitation, and their brains were rapidly removed. Selected brain structures (i.e., cortex, striatum and cerebellum) were isolated using brain matrix according to The Rat Brain Atlas, and were immediately subjected to SPME sampling. The entire experimental procedure, from the moment of anesthesia to insertion of the probes into the tissue, did not exceed 10 min.

### SPME sampling

Biocompatible SPME probes with C18 coating (extraction phase) were autoclaved at 121 °C for 20 min, and then were subjected to conditioning in a mixture of MetOH/water (50/50, *v/v*) for 30 min at 1000 rpm. Next, SPME probes were applied for the sampling of 3 brain regions (cerebellum, cerebral cortex and striatum) of young (1 month old), adult (3 months old), and old (24 months old) female and male rats. For the extraction of ECs from intact brain samples, pre-cut 4 mm length C18 probes were introduced into selected parts of brain for 30 min in static conditions. 3 SPME probes were inserted into cerebellum, 1 SPME probe into striatum and 1 SPME probe into cortex (Fig. S1). After the extractions had been completed, the probes were removed from the brain tissue, rinsed manually in water for 5 s to remove any loosely adhered biological matter, wiped with a paper tissue, and placed in empty vials. The vials were then snap frozen in dry ice for transportation to the lab. The probes remained stored in the empty vials at − 80 °C until LC–MS/MS analysis.

### Instrumental analysis

On the day of analysis, the SPME probes were defrosted and desorbed into 100 μL of a mixture of MetOH/IPA (50/50, *v/v*). The desorption was carried out for 30 min with agitation at 800 rpm in silanized inserts. Next, 10 ng/mL of AEA-d_11_ was added to the desorption solution at final concentration of 1 ng/mL. The extracts were injected into the LC–MS/MS system for targeted analysis of ECs. Liquid chromatographer coupled with triple quadrupole mass spectrometer LCMS-8060 (Shimadzu, Kyoto, Japan) was used for instrumental analysis. MS/MS analysis was performed in positive ionization mode under selected multiple reaction monitoring transitions as presented in Table S1. Experiments were performed using the Kinetex XB-C18 column (100 × 2.1 mm, 2.6 µm) (Phenomenex, USA). Autosampler (Nexera XR SIL-20AC XR) and column temperatures were set to 4 °C and 30 °C, respectively. The liquid chromatography was performed using mobile phase A composed of water with 0.1% FA and mobile phase B was composed of ACN with 0.1%FA. The following gradient was used for the separation of analytes: 0–0.1 min at 60% B; 0.1–6.0 min gradual increase to 95% B; 6.0–6.1 min of hold at 95% B; 6.1–8.0 min 60% B. The flow rate was set at 0.3 mL/min. Injection volume was 10 µL. The MS parameters were as follows: nebulizing gas flow rate: 3 L/min, heating gas flow rate: 10 L/min, interface temperature: 300 °C, desolvation line (DL) temperature: 250 °C, heating block temperature: 400 °C, drying gas flow rate: 7 L/min, interface voltage 4 kV. Data acquisition was performed with LabSolutions software version 5.97 (Shimadzu, Kyoto, Japan).

### Calibration curve and quantitation of ECs

The concentrations of ECs in the samples were calculated using the calibration curve that was prepared on the same day and analyzed in the same analytical run. Stock solutions of four ECs were prepared and used to generate standard calibration curves and to calculate the amounts of each EC extracted by SPME probes. Due to target compounds being present in surrogate brain homogenates, their quantitation with the use of matrix-matched external calibration approach was not possible. Hence, a calibration curve was constructed using phosphate buffered saline (PBS) (Cayman Chemical Company, Michigan, USA) containing a mixture of analytes at concentrations of AEA, NADA and 2-AGe ranging from 5 to 150 ng/mL and for 2-AG from 50 to 1500 ng/mL. Linearity of the described method was tested by the analysis of six series of PBS samples enriched with working standard solutions of ECs to obtain the analyte concentration at 5, 10, 25, 50, 100, and 150 ng/mL for AEA, NADA and 2-AGe, and at 50, 100, 250, 500, 1000, and 1500 ng/mL for 2-AG with the IS at concentration of 1 ng/mL added after the desorption step. The extractions of ECs from PBS were carried out with SPME probes identically to the probes used for the sampling from intact brain structures, and using the same extraction and desorption conditions as for the real samples as described in Sect. 2.3. The resulting weighted linear regression equations were applied for relative quantification of the amounts of ECs extracted from intact brain samples, yielding values of concentrations of the compounds of interest in brain. Additionally, a dilution study was performed to investigate whether the developed SPME-LC–MS/MS method can be applied to analyze samples at concentrations of 2-AG exceeding the upper limit of linearity. The additional investigations were provided for samples at 2000, 3000, 4000 and 5000 ng/mL concentrations of 2-AG, which were diluted by 10-folds in mixture of MetOH/IPA (50/50, *v/v*) and analyzed using the LC–MS/MS conditions described in Sect. 2.4. Thus, if the concentration of 2-AG in the sample was above the method’s linearity range, the tested sample was diluted by 10-folds in MetOH/IPA (50/50, *v/v*) prior to the LC–MS/MS analysis. The limit of detection (LOD) was defined as the signal of ECs obtained in PBS samples to the signal the signal obtained from blank samples (signal to noise level was 3:1), and the limit of quantification (LOQ) was defined as 10 times the signal obtained from blank samples.

### Data analysis

The chemometric assay of the autoscaled data sets describing the concentrations levels of 2-AG and AEA detected in three brain structures was conducted by HCA using the Ward’s method of agglomeration and Chebyshev distance measure, and also by PCA. For the multivariate analyses, Statistica 13.3 software (StatSoft, Tulsa, USA) was applied, and the calculations were performed on matrix data sets containing the concentrations of ECs (AEA or 2-AG) in specific parts of brain determined for tested eighteen rats, which were treated as the variables (18); description of the variables: gender (female (F) and male (M))_the type of ECs (AEA or 2-AG)_age (1 month (1 M); three months (3 M) and twenty four months (24 M)), whereas the structures of brain (cortex, striatum and cerebellum) were treated as the objects (3). The variables were standardized according to the formula:

$$\frac{xi-mean (x)}{\mathrm{SD}(x)}$$

where *x* is a sample variable from the set of all variables (*x*), and SD is standard deviation.

## Results and discussion

### Optimization of analytical protocol for the monitoring of ECs in brain tissue

As emphasized in several studies, the ECs are characterized by low stability in the samples, strong binding to plasma proteins, and also absorption to plastic ware, however, so far only few studies reported the use of silanized glass devices during the analysis of ECs in order to minimize their absorption to plastic devices (Dickens et al., 2020; Gachet et al., 2015). In addition, factors, such as enzymatic degradation, time consuming sample preparation and sample storage may affect the concentration of ECs (Luque-Córdoba et al., 2018; Zoerner et al., 2011). Hence, pre-analytical sample handling comprising adequate sampling and sample preparation is one of the most important steps in the analytical procedure aimed to precisely monitor the level and distribution of ECs in the sample matrix (Gurke et al., 2019; D. Kratz et al., 2022). In addition, the in vivo study conditions possessed inherent limitations that precludes the use of IS (e.g., pre-loading IS on the SPME fiber). Hence, IS can only be added to the sample extracts, thus correcting for instrumental analysis, but not for the extraction process.

**Fig. 1:**
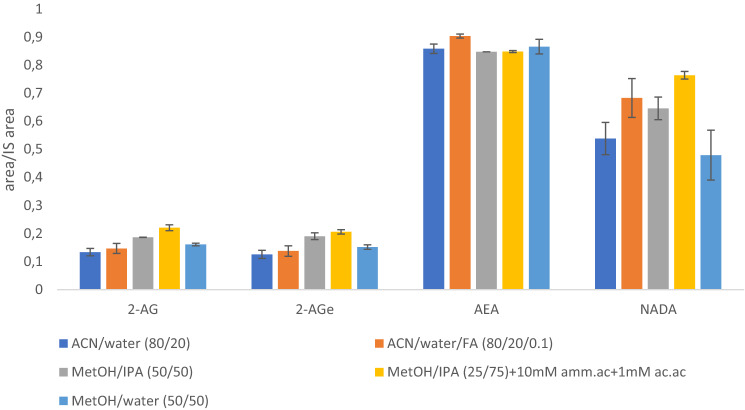
Optimization of SPME desorption step using C18 extraction phase and different combinations of desorption solvents (n = 5). The experiments were performed in initial conditions: 30 min extraction for PBS, 60 min desorption. Concentration of ECs in the samples was equal to 50 ng/mL and IS 1 ng/mL

In order to monitor the level of four ECs, i.e. AEA, 2-AG, NADA, and 2-AGe in different brain structures with the use of biocompatible SPME probes, it was necessary to optimize several parameters that affect the efficiency of the entire analytical procedure so as to facilitate in the future the extraction of ECs from various brain structures in the living organism (Lendor et al., 2019; Reyes-Garcés et al., 2019). The SPME probes used in this study were previously sterilized for potential application in in vivo studies and also this step additionally improves the performance of SPME probes as reported in previous studies (Roszkowska et al., 2018a, 2018b). The experiments were performed in silanized vials and inserts during the extraction of analytes from PBS and also during desorption step as it was shown that silanized glass ware improved the extraction efficiency for each analyte (Fig. S2). Due to the fact that ECs are hydrophobic compounds, the use of the C18 coating, according to earlier literature reports, is suitable for the isolation of different compounds possessing lipid structure, including ECs (Bogusiewicz et al., 2021; Roszkowska et al., 2019). As ECs are present in the samples at different levels (ng vs. µg), the optimization of the same conditions for compounds with such high diversity in the concentration range adds the complexity for their simultaneous analysis. However, based on the obtained results, it was observed that the performance of C18 fibers was much better for ECs present at trace levels in the matrices, i.e., AEA and NADA, whereas the extraction efficiency of C18 coating was significantly lower towards 2-AG, which however is abundant in real biological samples (Fig. 1). Other parameters that affect SPME method performance, such as extraction time, desorption time, and desorption solvent(s) were also optimized in this study. The solvents used for the desorption of the analytes from the SPME coating comprised mixtures of organic solvents (ACN, MetOH and IPA) and/or water used in various combinations and proportions with and without FA. As shown in Fig. 1, a mixture of MetOH/IPA (50:50, *v/v*) and also a mixture of MetOH/IPA (25/75, *v/v*) with addition of ammonium acetate and acidic acid presented the best recoveries among evaluated solvents for each analyte. Ultimately, a mixture MetOH/IPA (50:50, *v/v*) was selected for further analysis on ECs, which is in agreement with previous observations during the analysis of 2-AG and AEA analyzed in plasma samples (Acquaro Junior et al., 2019), and this mixture is also the most feasible during untargeted lipidomics studies (Bogusiewicz et al., 2021, 2022). In addition, the use of MetOH/IPA (50:50, v/v) mixture provided a carryover values lower than 2% for all compounds, which indicated that majority of extracted analytes were desorbed into the selected desorption mixture. Based on the conducted studies, the most efficient desorption time for all analytes was 30 min, however, NADA showed high instability on the SPME probe during the desorption (Fig. S3). The extraction time profile was evaluated by extracting ECs from intact brain matrix (Fig. S4). In vivo extraction time is mainly dictated by experimental limitations. In general, equilibrium extractions are desirable in SPME applications as they enable relatively simple calibration and also decreased irreproducibility arising from inaccuracies related to shorter extraction times. This is especially important in in vivo sampling, where the static extraction conditions impose longer equilibration times. As a proof of concept, the extraction time profile (5–30 min) was performed on intact brain structure (cerebellum), and it was confirmed that 30 min is suitable time for isolation of all analytes as shorter extraction time (5–20 min) prevents the isolation of two very labile ECs, i.e. NADA and 2-AGe from brain samples (Fig. S4). Shorter extraction time provided values of NADA and 2-AGe below their LODs. Longer (> 30 min) extraction times were not tested in this study as it could result in the morphological changes in the tissue due to dehydration, which can affect the extraction efficiency and ultimately the obtained results. Finally, the selected extraction time was 30 min performed under static conditions to mimic in vivo conditions as it represents a compromise between reasonably and sufficiently long sampling, providing sufficient recoveries and good reproducibility of measurements, while maintaining temporal resolution suitable for in vivo studies.

### Quantitation of ECs in selected brain regions

Although several approaches have been proposed for quantification of ECs in biological matrices, it still remains a challenge to analyze those endogenous molecules in solid matrices, such as brain tissue and hair samples due to endogenous nature of ECs, their high instability and rapid degradation/biotransformation (Gong et al., 2015; Voegel et al., 2021). However, the main difficulty still remains the lack of suitable (blank) matrix for preparation of calibration curves and further analysis of concentrations of those compounds in brain samples. One of previously reported approach comprised the addition of isotope-labeled standards to overcome the endogenous interferences and to generate calibration curves for ECs in brain matrix (Gong et al., 2015). However, such solution is not economically favorable and, based on the experiments preformed in this study, the recovery of ECs and its deuterated analogues differs, providing lower extraction efficiency of SPME probes for deuterated compounds isolated from brain homogenates. For quantitation of endogenous molecules from brain samples during SPME, 1% agarose gel or 2% agarose gel mixed with brain homogenate have been also proposed as surrogate matrix (Aslam et al., 2019; Lendor et al., 2019), however, in the case of labile ECs, such as NADA this approach could result in thermal degradation of this compound. It should be also emphasized that pooled brain samples used for the calibration curve preparation contain ECs, and what’s more, different batches of pooled sample matrix may generate different background levels. In consequence, as observed in this and previous studies, underestimation of the recovery occurs when subtracting a high background concentration of sample from low concentrations of added standards (Gong et al., 2015). Hence, due to lack of suitable brain matrix and the presence of high endogenous amounts of ECs in blank samples, the preparation of calibration curve was not possible with the matrix match approach, and hence for relative quantification of ECs in a neat solvent (PBS) was used for the preparation of the calibration curves.

The analysis of the calibration samples confirmed the developed method’s linearity over the entire concentration ranges for all analytes. Prepared calibration curves had a correlation coefficient (R^2^) of ≥ 0.99 (Table S2). The LOD were calculated using the calibration curve equation and were found to be 0.4 ng/mL for AEA, 1 ng/mL for NADA and 2-AGe, and 5 ng/mL for 2-AG (n = 6). Next, calculated calibration curves were used for analysis of the level and distribution of four ECs in cortex, cerebellum and striatum of nine female (F) and nine male (M) rats at different stages of development (1, 3 and 24 months old). The analysis of real rat brain samples indicated that two ECs, namely NADA and 2-AGE were isolated and detected in cortex samples, and also 2-AGe was detected in striatum samples, but the values were near the LODs, which suggests that SPME probes were unable to isolate measurable levels of those compounds from brain structures or those compounds were not present in indicated samples. In cortex, NADA was detected in female rats at all stages of development and also in 3 months old male rats, and what’s more, it was also present in striatum of 24 months old male and female rats (Fig. S5). In other groups, NADA was also identified, but its detection was not consistent, suggesting high lability of this EC, its fast biotransformation and/or its presence at ultra-trace levels, which is consistent with previous observations (Bobrich et al., 2020). Also, 2-AGe was not uniformly isolated from brain structures as this compound was detected only in the cerebellum of male rats (at all stages of development), and in cerebellum and striatum of females’ brain at each stage of development (Fig. S5). Those results are the first reports concerning the successful isolation and analysis of 2-AGe from brain structures as previous attempts to isolate this compound from brain samples have failed probably due to inadequate analytical protocol applied comprising tissue homogenization and utilization of organic solvent (ethyl acetate) for the extraction of this EC (Bobrich et al., 2020). In the case of 2-AG and AEA, these ECs were extracted from all analyzed brain regions. The result of semi-quantitative analysis of the level of AEA and 2-AG in each brain region of female and male rats at different stages of development are shown in Table S3. The concentrations of AEA found across the samples in analyzed brain structures of male and female rats at different stages of development ranged from 6.81 ± 6,89 to 68.04 ± 63.99 ng/g (Table S3). The concentrations of 2-AG were several orders of magnitude higher than all other analyzed compounds in all brain regions independently of gender of the rats. 2-AG concentrations (across areas) was measured well above the detection threshold in each brain section, and ranged from 1.09 ± 0.07 to 4.30 ± 1.09 µg/g in the analyzed brain samples. The obtained levels of AEA and 2-AG are in agreement with previously published report concerning the monitoring of the level of ECs in brain of rodents (Bobrich et al., 2020), where AEA and 2-AG could be reliably measured, however, in the above-mentioned study 2-AGe could not be detected in any of the analyzed samples, and NADA was detected only in some brain samples. Also, similar concentration ranges of AEA and 2-AG were obtained in other studies analyzing the levels of those two ECs in different brain regions (prefrontal cortex, hippocampus and hypothalamus) of tumor-bearing mice (Gong et al., 2015), in different rat models of depression (Smaga et al., 2017), and in striatum ipsilateral and contralateral of rat brain samples from an animal model of Parkinson’s disease (Oliveira et al., 2021).

Overall, the results obtained in this study show promise in the application of SPME probes to further understanding the relationship between the levels of particular ECs and their role in the signaling within different brain regions. It should be emphasized that the ultimate goal of this study was to optimize the SPME protocol suitable for in vivo monitoring of the level of particular ECs in different brain regions, however, several limitations related to analysis of endogenous compounds in brain tissue still need further improvements. Moreover, the lack of the ability to use IS correction for extraction of ECs during in vivo studies (e.g. any compound can be neither administered nor loaded in the probe as IS) makes this analysis challenging as AEA-d_11_ (IS) was added to the desorption solution only for the correction of LC–MS/MS analysis. Hence, more studies are needed to capture unstable and transient ECs and to depict their presence and levels in the brain. In this respect, in vivo SPME sampling can provide real information about participation of those compounds in biochemical processes ongoing in the living system.

### Analysis of the level of AEA and 2-AG in selected brain structures in age- and gender-dependent approach

**Fig. 2:**
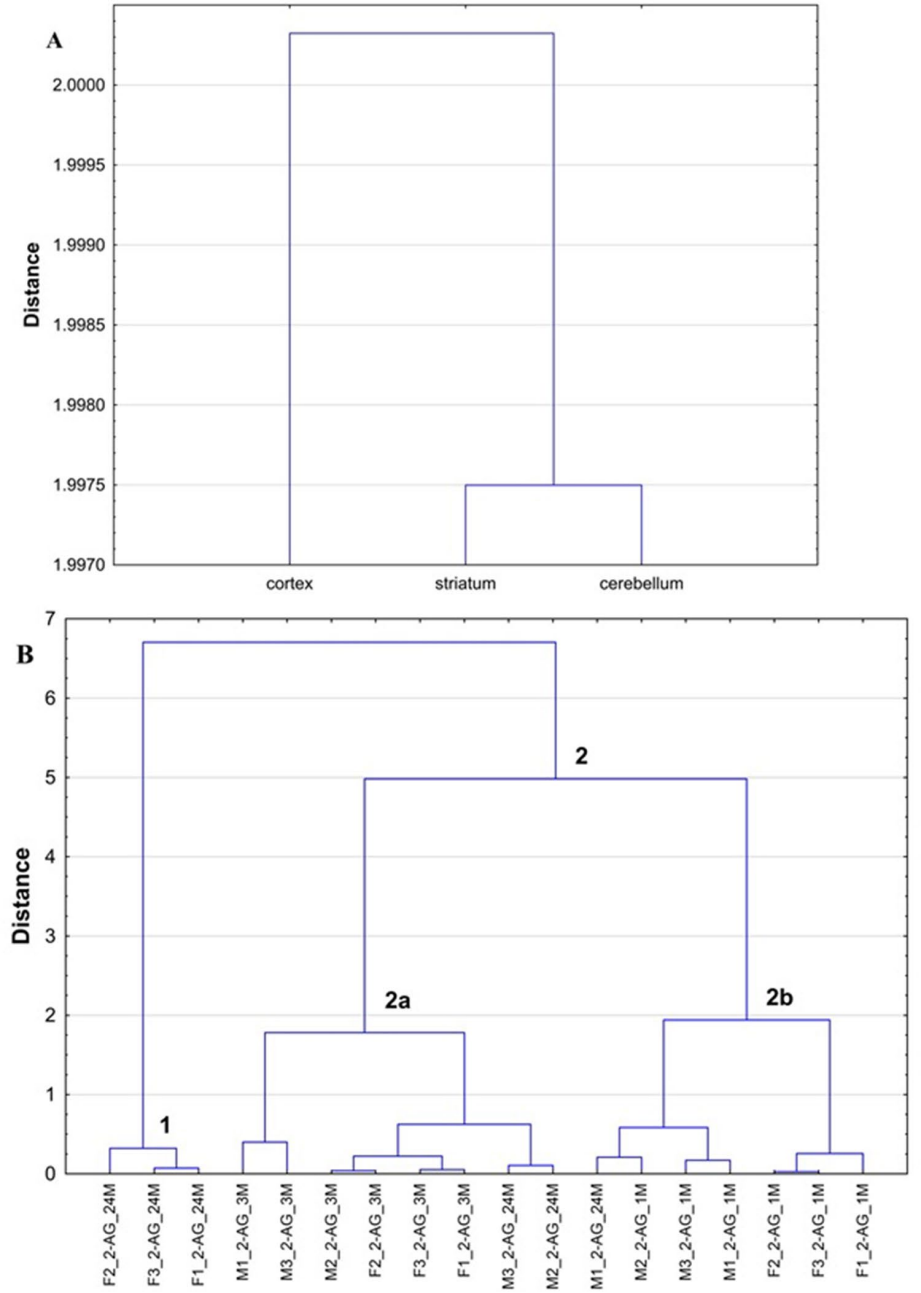
Dendrograms calculated by HCA on the basis of the established 2-AG levels in analyzed brain structures for male and female rats at different stage of development (1 month old, 3 months old and 24 months old) (A—objects, B—variables)

**Fig. 3:**
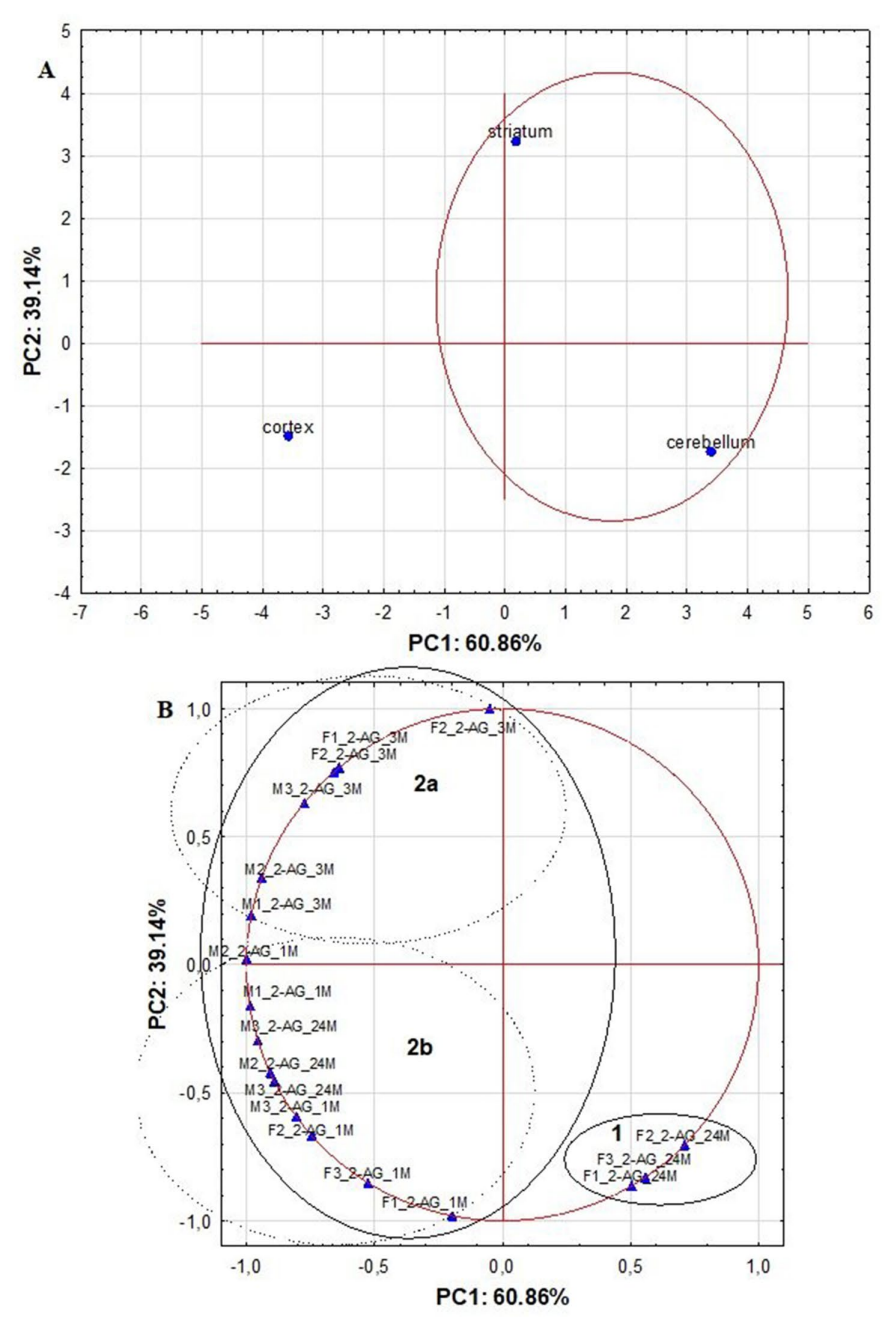
The score and loading plots based on the autoscaled 2-AG results for analyzed rat brain structures picturing the objects and the variables in two-dimensional space

**Fig. 4:**
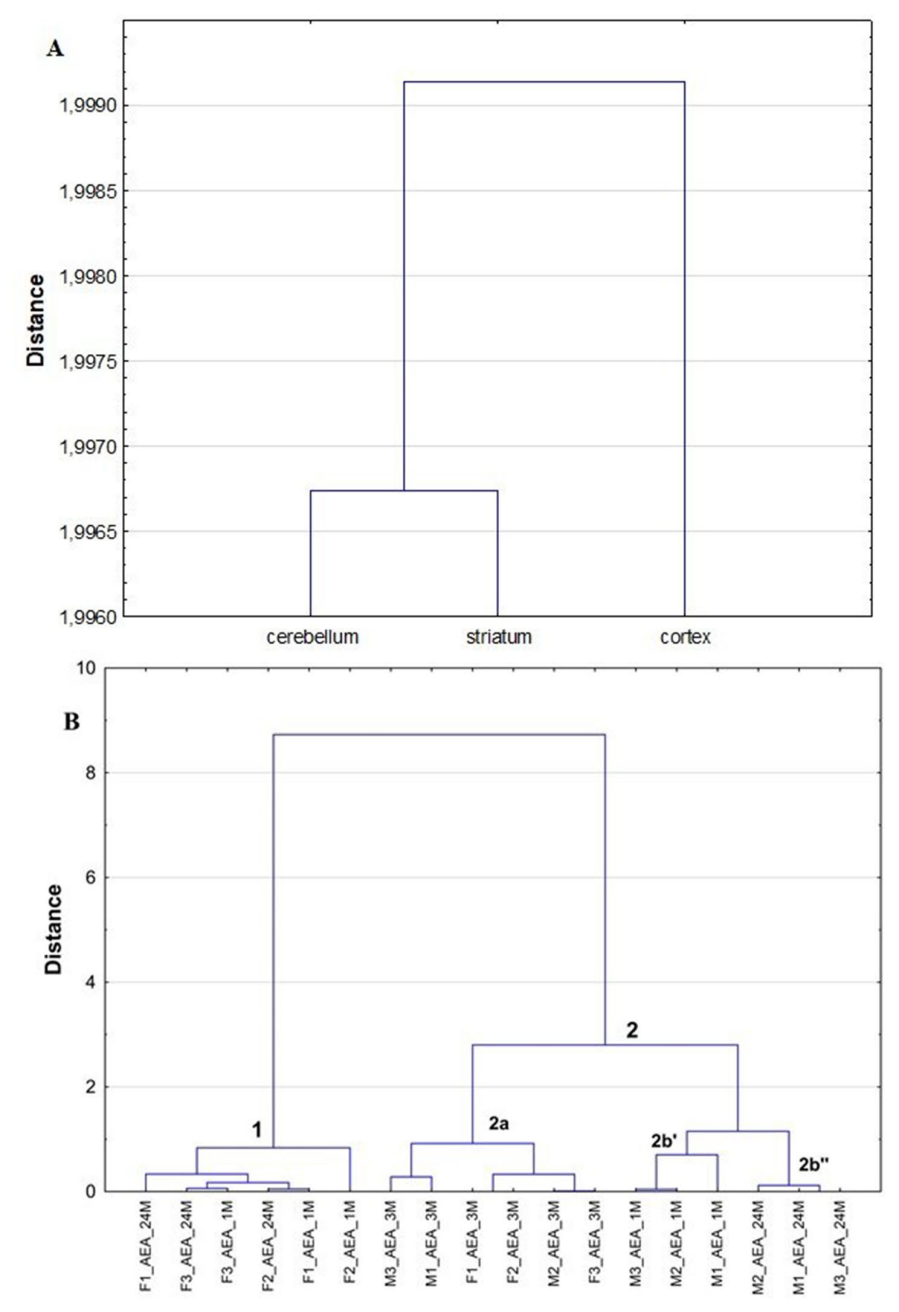
Dendrograms calculated by HCA on the basis of the established AEA levels in analyzed brain structures for male and female rats at different stage of development (1 month old, 3 months old and 24 months old) (A—objects, B—variables)

**Fig. 5:**
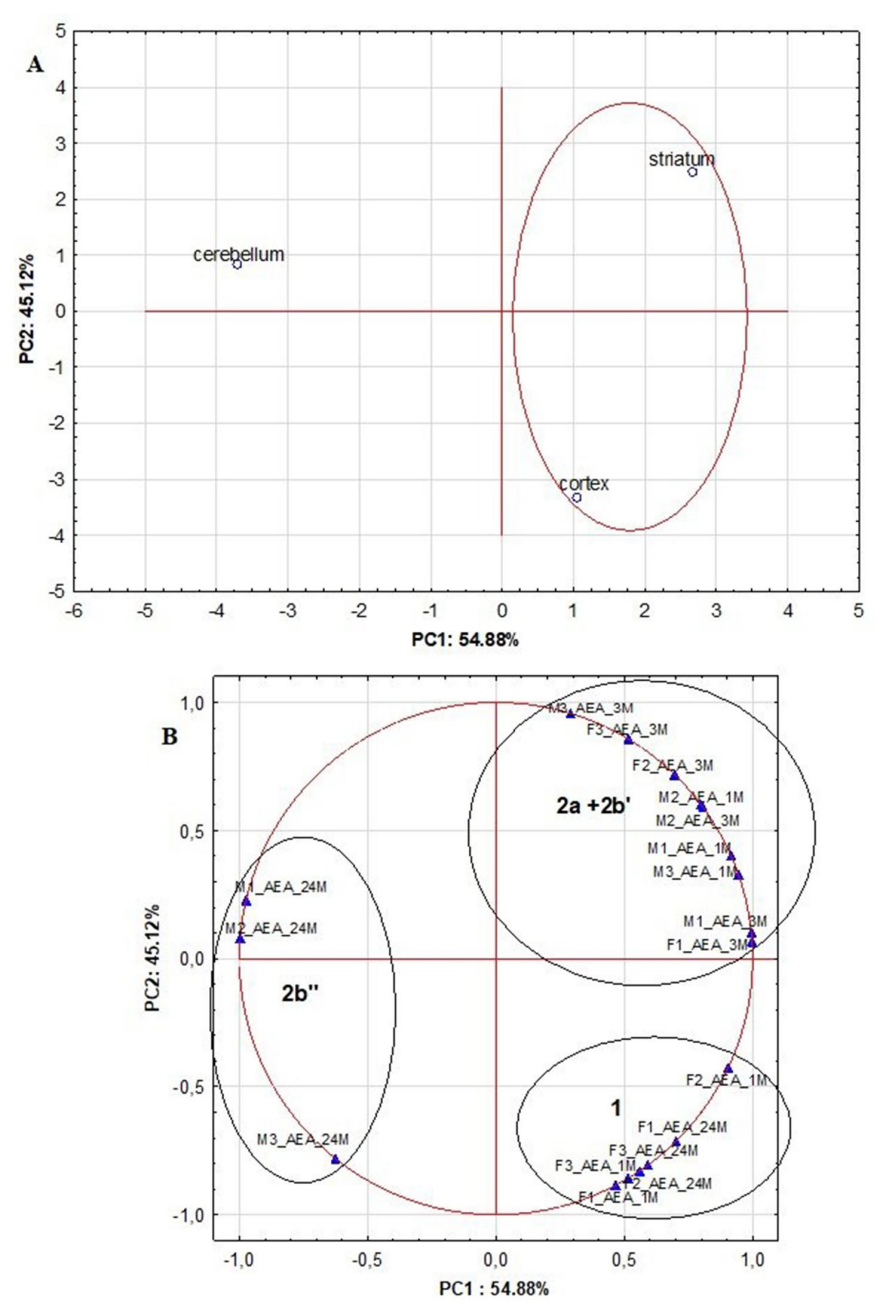
The score and loading plots based on the autoscaled AEA results for analyzed rat brain structures picturing the objects and the variables in two-dimensional space

The datasets describing the levels of 2-AG and AEA in the analyzed structures of rat brain were also evaluated by the HCA and PCA multivariate analyses to provide insights of the relationship between the level of those ECs and particular brain regions. Dendrograms and PCA scores and loadings plots corresponding to the first two PCs calculated on the basis of detected levels of 2-AG in three rat brain structures of 18 tested rats are shown in Fig. 2 and Fig. 3, whereas for AEA are shown in Fig. 4 and 5, respectively. In HCA calculations based on data describing the levels of 2-AG, cerebellum and striatum were included into the same cluster, whereas cortex was located as outlier on the left of the dendrogram (Fig. 2A). In fact, the levels of 2-AG in cortex of male and female rats, except for those found for 1 and 24 months old females, were lower than those measured in cerebellum and striatum (Table S3). Moreover, the HCA results for the observations showed that only F1_2-AG_24M, F2_2-AG_24M and F3_2-AG_24M were located in cluster 1, while most variables were positioned in cluster 2 (Fig. 2B). For the rats included in cluster 1, the analyzed ECs was determined at the highest level in cortex (3.88 ± 0.15 µg/g), while these values were lower in striatum (2.56 ± 0.77 µg/g) and cerebellum (3.36 ± 0.84 µg/g) (Table S3). Moreover, the structure of cluster 2 created by HCA for the variables indicates that comparable differences in the concentrations of 2-AG were found in three tested brain structures for 3 month old rats regardless the gender (these rats are included to subcluster 2a). In fact, for those groups of rats, the levels of 2-AG in female and male rats were higher in striatum (2.48 ± 0.17 µg/g and 2.66 ± 0.17 µg/g) and lowers in cerebellum (2.35 ± 0.84 µg/g and 2.85 ± 0.61 µg/g) in respect to the levels of 2-AG found for the 1 month old female and male rats positioned in subcluster 2b (striatum: 2.18 ± 0.70 µg/g and 2.19 ± 0.79 µg/g; cerebellum: 2.92 ± 0.41 µg/g and 3.94 ± 0.14 µg/g). Two 24 months old male rats (M2 and M3) having 2-AG levels of 2.16 µg/g and 2.82 µg/g in striatum, and 4.15 µg/g and 4.49 µg/g in cerebellum, respectively, were also included to cluster 2a. On the other hand, 24 months old male rat (M1) was included to subcluster 2b. For this rat, a level of 2-AG was higher in striatum (3.88 µg/g) and comparable in cerebellum (4.28 µg/g) in respect to others included to this group.

Of note, based on the PCA results, comparable relationships between the objects and the variables was found as indicated by the HCA, except for the classification of 24 months old male rats. In the case of PCA calculations, the first two PCs explained 100% of the data variability (Fig. 3). Moreover, the variances of the variables describing the concentration levels of 2-AG for 1 month old and 3 months old rat males as well as 3 months old and 24 months old male rats were mainly explained by the PC1 (60.85%), whereas the variability of the concentrations of analyzed ECs established for 1 month old and 24 months old female rats were mainly explained by the PC2 (39.14%). The PCA scores plot presented in Fig. 3A also showed the cortex as the outlier, while striatum and cerebellum were positioned together on the right of the plot. Moreover, the PCA loadings plot positioned a group of 24 months old female rats on the right side of the plot in cluster 1, while other variables were located on the left side of the plot into cluster 2 (Fig. 3B). The localization of the variables within cluster 2 was also comparable to those observed on the dendrogram calculated by the HCA. The differences in the levels of ECs measured in 3 months old female and male rats in respect to the levels found in 1 month old male and female rats was also indicated by distance between these variables. Those observations were located in two subclusters (2a and 2b, respectively) on the left of side the plot. The group of 24 months old male rats was included in subcluster 2b, and this localization was different than observed in HCA, where M1_2-AG_24M was included to subcluster 2b, but M2_2-AG_24M and M3_2-AG_24M were positioned in subcluster 2a. This indicates that slightly different relationships between the variables were established by PCA and those calculated by HCA.

Other relationships between the objects and the variables established by HCA and PCA for the levels of AEA calculated in the samples obtained from all analyzed groups of female and male rats were used in chemometric calculations. For this data set, the HCA positioned cerebellum as an outlier, while striatum and cortex were located together on the right of the dendrogram (Fig. 4A). It is in accordance with detected levels of AEA in three brain structures of 18 tested rats; low AEA levels were found in cerebellum (from 6.81 ± 6.89 ng/g for 3 months old male rats to 22.40 ± 12.05 ng/g for 24 months old female rats), while the concentrations of AEA were from 22.04 ± 5.08 ng/g for 24 months old female rats to 68.04 ± 63.99 ng/g for 3 months old female rats in striatum, and from 10.81 ± 2.36 ng/g for 3 months old male rats to 54.81 ± 67.00 ng/g for 1 month old female rats in cortex, respectively (Table S3). Thus, HCA confirmed different distribution of AEA in respect to 2-AG in analyzed brain structures in the analyzed rats (Fig. 4A vs. Figure 3B). Moreover, HCA results located the groups of 24 and 1 month old female rats in cluster 1 (Fig. 4B). For those rats, comparable AEA levels were measured in striatum, while the concentrations of this compound were higher in cortex (Table S3). The HCA also created cluster 2, which structure indicated that the levels of AEA slightly varied among three tested brain structures. Comparable distribution of AEA (the highest values in striatum, moderate in cortex and the lowest in cerebellum) were found for 3 months old male and female rats (Table S3); those rats were included to subcluster 2a. Also, comparable distribution of AEA in cortex and striatum to observed for the rats included in subcluster 2a was found for 1 month old male rats positioned in subcluster 2b’, but for them the concentration of AEA in cerebellum was higher taking into account its levels in striatum and cortex. In the case of 24 months old males, the levels of AEA measured in cerebellum were also relatively high (19.4 ± 10.22 ng/g), but additionally this ED was found in comparable concentration in striatum and cortex (35.68 ± 12.3 and 33.02 ± 5.33 ng/g, respectively) (Table S3); hence, this group of rats was positioned by HCA in subcluster 2b”.

The relationships between the objects and the variables established by the HCA were also confirmed by PCA results (Fig. 5). Therefore, PCA calculations allows to explain 100% of the data variability on PCA scores and loadings plots where 54.88% of the variances of the variables was established by the first PC and 45.12% by the second PC, respectively. Additionally, the variability of the variables showing the levels of AEA for 1 month old and 3 months old male rats and for 3 months old female rats were mainly explained by the PC1, while the variances in the level of AEA in 1 month old and 24 months old female rats were mainly explained by the PC2. Thus, according the PCA scores plot (Fig. 5A), cerebellum was located as outlier on the left side of the plot, while striatum and cortex were positioned together on the right part of the plot. Moreover, PCA calculations positioned the same observations into cluster 1, while in the cluster positioned upper side on the right part of the PCA loadings plot were located all observations positioned by HCA in subclusters 2a and 2b’. However, PCA much stronger highlighted the differences in the distribution of AEA in 24 months old male rats, which were positioned on the left of the PCA loadings plot in cluster 2b”. It confirms that for most variables comparable relationships between them were established by this chemometric tool as found by the HCA (Fig. 4B).

In summary, HCA and PCA assays allowed to show the relationships between the levels of 2-AG and AEA measured in three structures of rat brain depending on the age and gender of analyzed rats. These chemometric data showed that different levels of 2-AG were established in cortex (mainly low) in respect to those observed in cerebellum and striatum (mainly high or middle), while for AEA, the levels of this EC were high and middle in cortex and striatum, but were low in cerebellum. Additionally, the highest differences in the levels of 2-AG were found between 24 months old female rats and other analyzed groups of rats. It was also observed that 3 months old rats (regardless the gender) had comparable distribution of 2-AG and also AEA in cortex, striatum and cerebellum, whereas the differences in the level of particular ECs in each brain region were higher for younger and older male and female rats. Thus, the obtained results suggest that the activity of particular ECs in each brain region differs, which may also affect the functioning on particular brain structures.

Overall, the observed changes in the distribution of particular ECs seemed to be associated with specific brain structures and also with the factors related to the gender of rats and stage of brain maturation. In the study performed by Smaga et. on animal models of depression in 8–9 months old male rats, the level of AEA and 2-AG differed depending on the brain structure (Smaga et al., 2017). As confirmed in other studies, the level of neuroprotective EC, i.e. 2-AG was decreased in the hippocampus of old mice, which may contribute to the progression of brain ageing (Piyanova et al., 2015). However, factors, such as diet, stress conditions or cancer may also affect the level of ECs in plasma and brain samples, which adds the complexity into the analysis of those endogenous molecules (Aslam et al., 2019; Gong et al., 2015; Pati et al., 2018). Hence, introduction of SPME probes for in vivo sampling opens new possibilities in the analysis of those labile tissue components, and provide new tools for precise monitoring of the level of ECs in different brain regions in the living organisms.

## Conclusion

In this study, SPME sampling utilizing biocompatible C18 probes along with LC–MS/MS analysis was applied as a novel strategy to monitor trace levels of ECs, including AEA, 2-AG, NADA and 2-AGe in selected brain regions (cerebellum, cortex and striatum) of young, adult and old female and male rats. The optimized SPME-LC–MS/MS method facilitated isolation of ECs from intact brain samples without the need for tissue homogenization. Due to its simplicity and low invasiveness, SPME probes provide significant advantages during the isolation of even trace levels of labile and short-live endogenous compounds in intact solid tissues, however, complete quantitation methodology for analysis of ECs in brain structures still needs improvements. Statistically significant differences in the level of particular ECs were observed between young, adult and old rats, and also between female and male rats. The HCA and PCA results revealed the relationships between the levels of 2-AG and AEA in three brain structures of male and female rats. These chemometric calculations indicated that the distribution of 2-AG and AEA in cortex, striatum and cerebellum is different and also is dependent on the gender and age of rats. Thus, the activity of each brain structure at different stages of brain development of male and female rats may be different, depending on the level and distribution of particular ECs. In addition, two highly unstable ECs that are present at trace levels in brain samples, namely NADA and 2-AGe, were successfully isolated from intact brain samples with the use for SPME probes. These preliminary studies concerning direct monitoring of level of ECs in intact brain samples are a starting point for next SPME studies of complex biochemical interactions and behavior of ECs in different brain regions, and are the basis for further research including the analysis of these important endogenous molecules in the living organisms in physiological and pathological conditions.

## Supplementary Information

Below is the link to the electronic supplementary material.

Supplementary file2 (PDF 309 KB)—. SPME extraction from intact brain regions with the use of 4 mm length C18 probes. 3 SPME probes were inserted into cerebellum (upper part), 1 SPME probe into cortex (middle part) and 1 SPME probe into striatum (lower part) of each analyzed groups of rats.

Supplementary file2 (PDF 45 KB)—The extraction efficiency of ECs at 50 ng/mL concentration in PBS with the use of C18 probes. The extractions from regular and silanized glass vials were tested.

Supplementary file3 (PDF 50 KB)—Optimization of desorption time profile (DTP) of analyzed ECs from PBS during SPME analysis. The extraction of ECs (c=50 ng/mL) was performed for 30 min. extraction for PBS, desorption was performed from 10 min to 90 min. Experiments were performed in triplicates for each time point.

Supplementary file4 (PDF 59 KB)—Optimization of extraction time profile (ETP) of analyzed ECs from intact brain structure (cerebellum) during SPME. The extraction of ECs was performed from 5 min to 30 mi. Desorption of analytes was performed for 30 min into 100 μL of a mixture of MetOH/IPA (50/50, v/v) and AEA-d11 IS at 1 ng/mL concentration. Experiments were performed in triplicates for each time point.

Supplementary file5 (PDF 54 KB)—Analysis of the level and distribution of NADA and 2-AGe in three brain structures in 1 month old, 3 months old and 24 months old rats (females and males). The analytes were isolated from intact brain samples with the use of autoclaves C18 SPME probes during static extraction for 30 min. Desorption of analytes was performed for 30 min into 100 μL of a mixture of MetOH/IPA (50/50, v/v) and AEA-d11 IS at 1 ng/mL concentration.

Supplementary file6 (DOCX 23 KB)

## Data Availability

All data generated or analyzed during this study are included in this published article and its supplementary information files. Processed data can be available upon request.
