# Supplementary figures and images for: Monitoring of age- and gender-related alterations of endocannabinoid levels in selected brain regions with the use of SPME probes
Source: Metabolomics. 2023 Apr 12;19(4):40. doi: 10.1007/s11306-023-02007-9 (PMC10097736; doi:10.1007/s11306-023-02007-9)

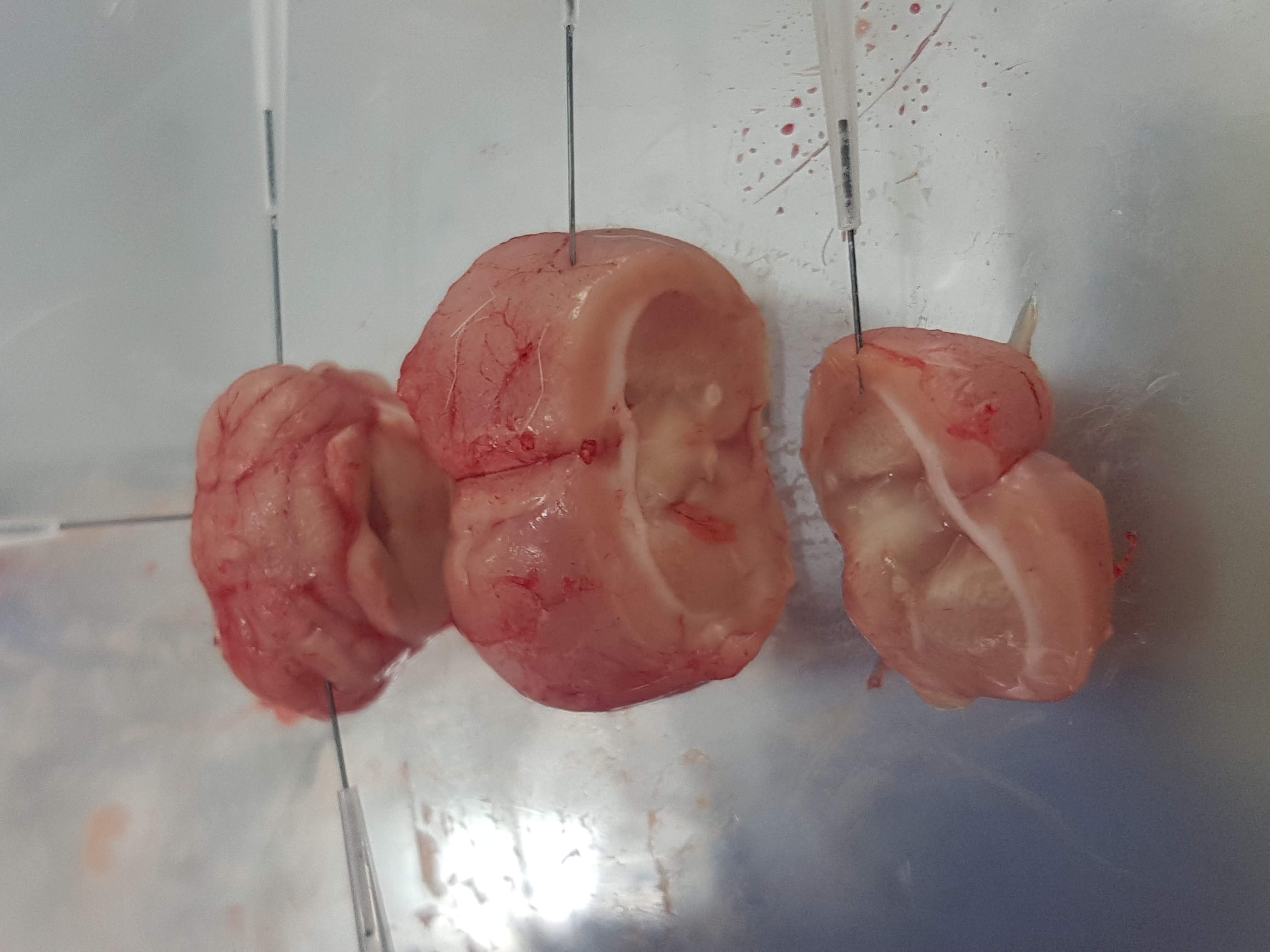

Supplement: Supplementary file 1 — Supplementary file2 (PDF 309 KB)—. SPME extraction from intact brain regions with the use of 4 mm length C18 probes. 3 SPME probes were inserted into cerebellum (upper part), 1 SPME probe into cortex (middle part) and 1 SPME probe into striatum (lower part) of each analyzed groups of rats. [file 11306_2023_2007_MOESM1_ESM.jpg]

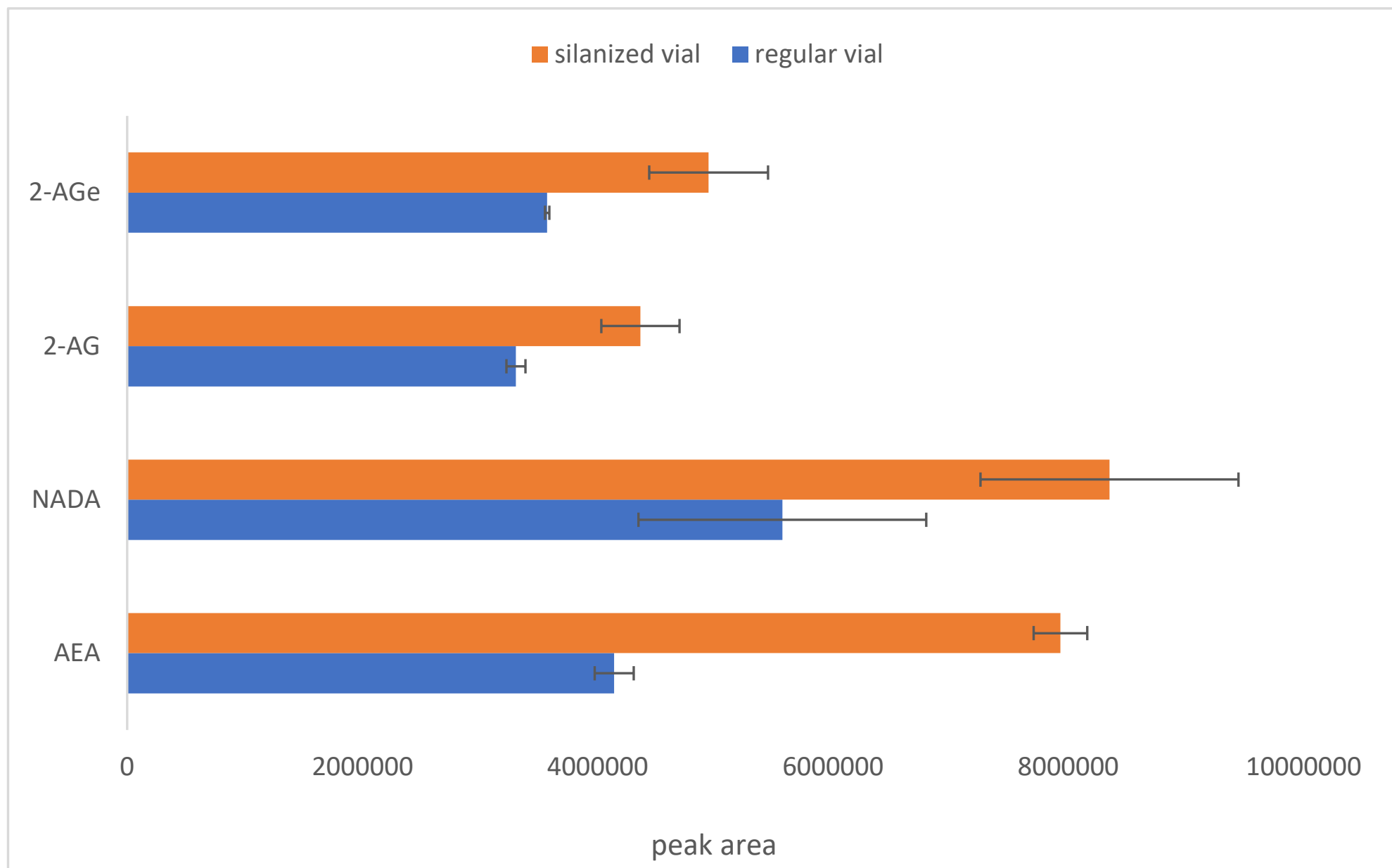

Supplement: Supplementary file 2 — Supplementary file2 (PDF 45 KB)—The extraction efficiency of ECs at 50 ng/mL concentration in PBS with the use of C18 probes. The extractions from regular and silanized glass vials were tested. [file 11306_2023_2007_MOESM2_ESM.pdf]

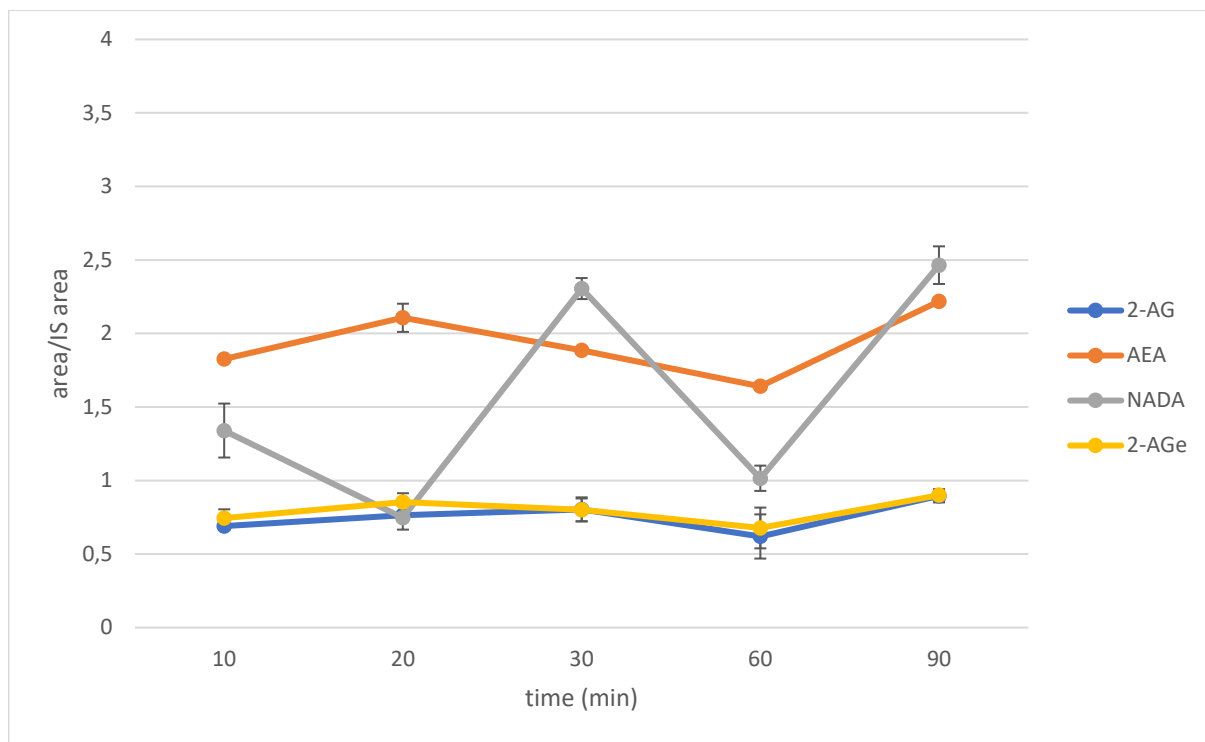

Supplement: Supplementary file 3 — Supplementary file3 (PDF 50 KB)—Optimization of desorption time profile (DTP) of analyzed ECs from PBS during SPME analysis. The extraction of ECs (c=50 ng/mL) was performed for 30 min. extraction for PBS, desorption was performed from 10 min to 90 min. Experiments were performed in triplicates for each time point. [file 11306_2023_2007_MOESM3_ESM.pdf]

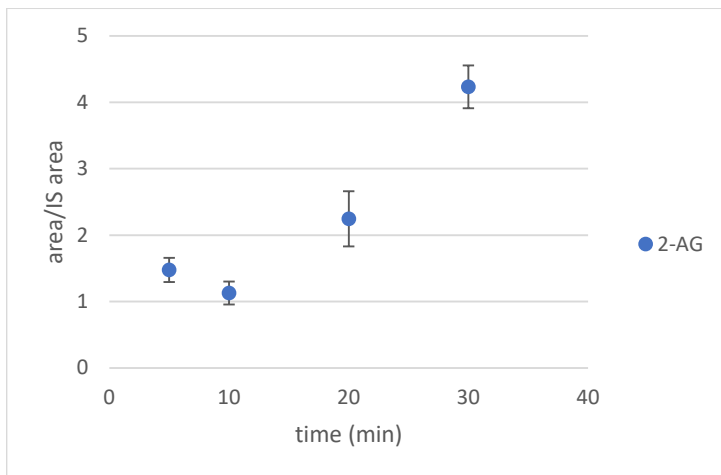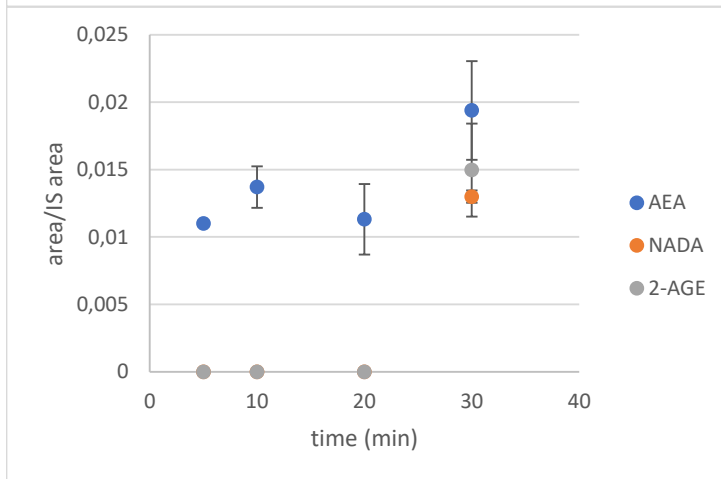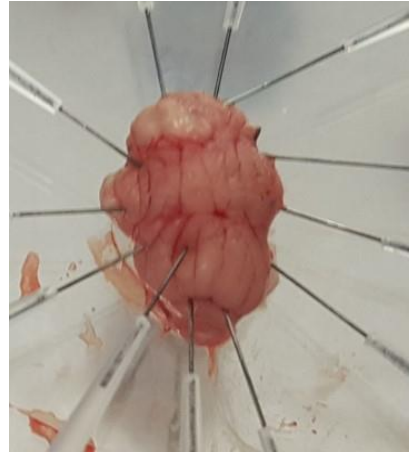

Supplement: Supplementary file 4 — Supplementary file4 (PDF 59 KB)—Optimization of extraction time profile (ETP) of analyzed ECs from intact brain structure (cerebellum) during SPME. The extraction of ECs was performed from 5 min to 30 mi. Desorption of analytes was performed for 30 min into 100 μL of a mixture of MetOH/IPA (50/50, v/v) and AEA-d11 IS at 1 ng/mL concentration. Experiments were performed in triplicates for each time point. [file 11306_2023_2007_MOESM4_ESM.pdf]

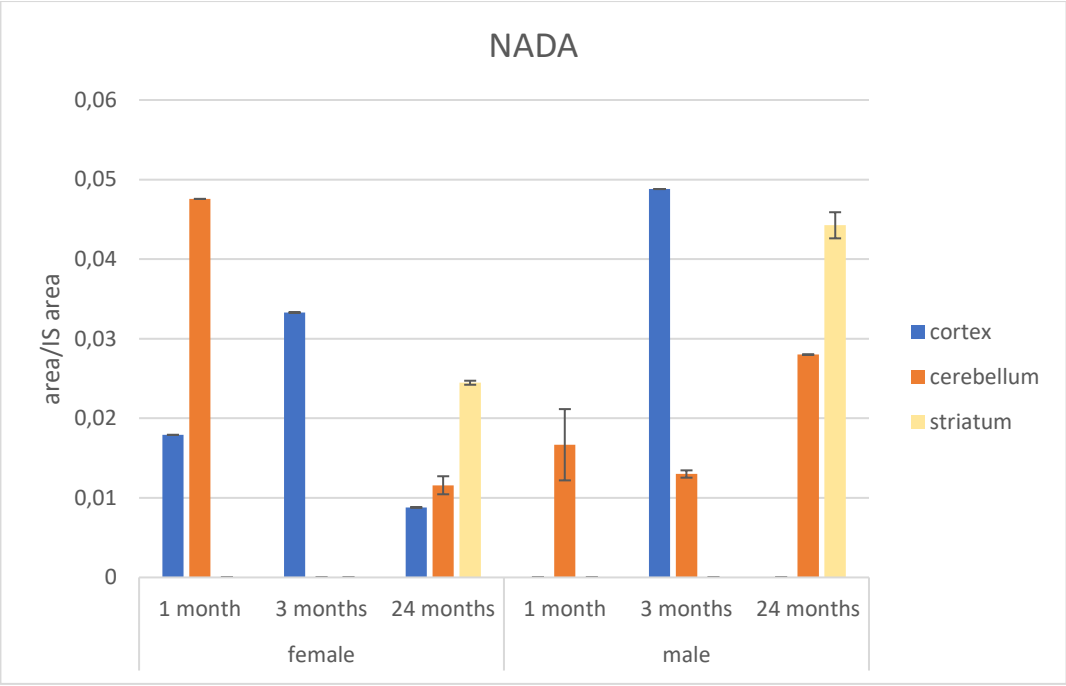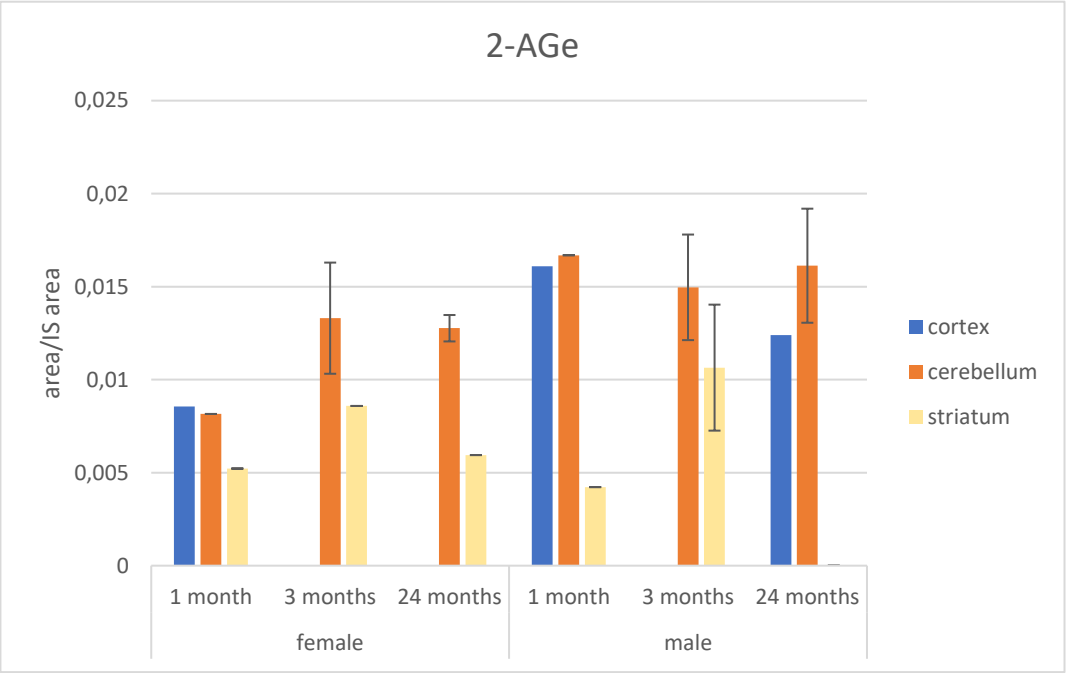

Supplement: Supplementary file 5 — Supplementary file5 (PDF 54 KB)—Analysis of the level and distribution of NADA and 2-AGe in three brain structures in 1 month old, 3 months old and 24 months old rats (females and males). The analytes were isolated from intact brain samples with the use of autoclaves C18 SPME probes during static extraction for 30 min. Desorption of analytes was performed for 30 min into 100 μL of a mixture of MetOH/IPA (50/50, v/v) and AEA-d11 IS at 1 ng/mL concentration. [file 11306_2023_2007_MOESM5_ESM.pdf]
