# Supplementary material for: Monitoring of age- and gender-related alterations of endocannabinoid levels in selected brain regions with the use of SPME probes
Source: Metabolomics. 2023 Apr 12;19(4):40. doi: 10.1007/s11306-023-02007-9 (PMC10097736; doi:10.1007/s11306-023-02007-9)
Supplement: Supplementary file 6 — Supplementary file6 (DOCX 23 KB) [file 11306_2023_2007_MOESM6_ESM.docx]

**Monitoring of age- and gender-related alterations of endocannabinoid levels in selected brain regions with the use of SPME probes**

Anna Roszkowska^1*^, Ilona Klejbor^2^, Joanna Bogusiewicz^3^, Alina Plenis^4^, Barbara Bojko^3^, Katarzyna Kowalik^1^, Janusz Moryś^5^, Tomasz Bączek^1^

^1^Department of Pharmaceutical Chemistry, Medical University of Gdańsk, Gdańsk, Poland

^2^Department of Anatomy, Jan Kochanowski University, Institute of Medical Sciences, Kielce, Poland

^3^Department of Pharmacodynamics and Molecular Pharmacology, Collegium Medicum in

Bydgoszcz, Nicolaus Copernicus University in Toruń, Bydgoszcz, Poland

^4^Department of Analytical Chemistry, Medical University of Gdańsk, Gdańsk, Poland

^5^Department of Normal Anatomy, Pomeranian Medical University, Szczecin, Poland

**Captions of Figures and Tables**

**Table S1**. Monitored precursor–product ion(s) transitions and retention time for particular ECs.

| **Compound** | **Precursor m/z** | **Product m/z** | **Collision energy (eV)** | **Transition selected for quantitative analysis** | **Retention time (min)** |
| --- | --- | --- | --- | --- | --- |
| 2-AG | 379.20 | 287.25 | -15 | + | 4.32 |
|  | 379.20 | 269.00 | -18 |  |  |
| 2-AGe | 365.20 | 273.10 | -12 | + | 4.81 |
|  | 365.20 | 121.00 | -23 |  |  |
| AEA-d_11_ | 359.40 | 62.10 | -20 | + | 3.69 |
|  | 359.40 | 214.10 | -15 |  |  |
| AEA | 348.20 | 62.15 | -20 | + | 3.75 |
|  | 348.20 | 287.10 | -13 |  |  |
| NADA | 440.20 | 137.00 | -27 | + | 4.22 |
|  | 440.20 | 153.95 | -18 |  |  |
|  | 440.20 | 119.15 | -40 |  |  |

**Table S2.** Parameters of calibration curves used for semi-quantitation of ECs in intact brain structures using SPME probes.

| compound | slope | intercept | R^2^ |
| --- | --- | --- | --- |
| 2-AG | 0,0347 | - 0,715 | 0,999 |
| AEA | 0,0083 | 0,0381 | 0,999 |
| NADA | 0,0154 | - 0,0455 | 0,9969 |
| 2-AGe | 0,0038 | - 0,0119 | 0,9957 |

**Table S3.** Semi-quantitative analysis of the level of 2-AG and AEA in 3 different brain regions of female and male rats at different stages of development. The analytes were extracted from intact brain tissue samples with the use of SPME probes.

| **AEA** (ng/g) | | | | | | |
| --- | --- | --- | --- | --- | --- | --- |
|  | 1 month old | | 3 months old | | 24 months old | |
|  | female (n=3) | male (n=3) | female (n=3) | male (n=3) | female (n=3) | male (n=3) |
| cortex | 54.81±67.0 | 19.6±3.13 | 23.92±14.77 | 10,81±2,36 | 34.89±2.12 | 33.02±5.33 |
| striatum | 24.84±16.55 | 29.15±1.14 | 68.04±63.99 | 23,27±2,37 | 22.04±5.08 | 35.68±12.3 |
| cerebellum | 8.91±3.93 | 12.94±4.46 | 12.72±7.25 | 6,81±-6,89 | 22.40±12.05 | 19.4±10.22 |
| **2-AG** (µg/g) | | | | | | |
|  | 1 month old | | 3 months old | | 24 months old | |
|  | female (n=3) | male (n=3) | female (n=3) | male (n=3) | female (n=3) | male (n=3) |
| cortex | 2.34±0.48 | 1.48±0.44 | 1.09±0.07 | 1.62±0.74 | 3.88±0.15 | 1.73±0.81 |
| striatum | 2.18±0.7 | 2.19±0.79 | 2.48±0.17 | 2.66±0.17 | 2.56±0.77 | 2.98±1.81 |
| cerebellum | 2.92±0.41 | 3.94±0.14 | 2.35±0.84 | 2.85±0.61 | 3.36±0.84 | 4.3±1.09 |

**Fig. S1.** SPME extraction from intact brain regions with the use of 4 mm length C18 probes. 3 SPME probes were inserted into cerebellum (upper part), 1 SPME probe into cortex (middle part) and 1 SPME probe into striatum (lower part) of each analyzed groups of rats.

**Fig. S2.** The extraction efficiency of ECs at 50 ng/mL concentration in PBS with the use of C18 probes. The extractions from regular and silanized glass vials were tested.

**Fig. S3.** Optimization of desorption time profile (DTP) of analyzed ECs from PBS during SPME analysis. The extraction of ECs (c=50 ng/mL) was performed for 30 min. extraction for PBS, desorption was performed from 10 min to 90 min. Experiments were performed in triplicates for each time point.

**Fig. S4.** Optimization of extraction time profile (ETP) of analyzed ECs from intact brain structure (cerebellum) during SPME. The extraction of ECs was performed from 5 min to 30 mi. Desorption of analytes was performed for 30 min into 100 μL of a mixture of MetOH/IPA (50/50, v/v) and AEA-d_11_ IS at 1 ng/mL concentration. Experiments were performed in triplicates for each time point.

**Fig. S5.** Analysis of the level and distribution of NADA and 2-AGe in three brain structures in 1 month old, 3 months old and 24 months old rats (females and males). The analytes were isolated from intact brain samples with the use of autoclaves C18 SPME probes during static extraction for 30 min. Desorption of analytes was performed for 30 min into 100 μL of a mixture of MetOH/IPA (50/50, v/v) and AEA-d_11_ IS at 1 ng/mL concentration.

—
